# Hybrid Microporous Polymeric Materials with Outstanding Permeability and Increased Gas Transport Stability: PTMSP Aging Prevention by Sorption of the Polymerization Catalyst on HCPS

**DOI:** 10.3390/polym13121922

**Published:** 2021-06-09

**Authors:** Georgy Golubev, Danila Bakhtin, Sergey Makaev, Ilya Borisov, Alexey Volkov

**Affiliations:** A.V. Topchiev Institute of Petrochemical Synthesis RAS, 29 Leninsky Prospekt, 119991 Moscow, Russia; db2@ips.ac.ru (D.B.); makaev@ips.ac.ru (S.M.); boril@ips.ac.ru (I.B.); avolkov@ips.ac.ru (A.V.)

**Keywords:** gas separation, PTMSP, aging, hyper-crosslinked polystyrene, mixed matrix membrane, polymerization catalyst, polymer purification

## Abstract

The influence of hyper-crosslinked polystyrene (HCPS) Macronet^TM^ MN200 on the gas transport properties and aging of the highly permeable glassy polymer poly(1-trimethylsilyl-1-propyne) (PTMSP) was studied and analyzed in detail. The gas transport characteristics of dense PTMSP membranes containing 0–10.0 wt % HCPS were studied. It was shown that the introduction of a small amount of HCPS into the PTMSP matrix led to a 50–60% increase of the permeability coefficients of the material for light gases (N_2_, O_2_, CO_2_) and slowed down the deterioration of polymer transport properties over time. The lowest reduction in gas permeability coefficients (50–57%) was found for PTMSP containing HCPS 5.0 wt % after annealing at 100 °C for 300 h. It was found that HCPS sorbed residues of tantalum-based polymerization catalyst from PTMSP. In order to investigate the influence of catalysts on transport and physical properties of PTMSP, we purified the latter from the polymerization catalyst by addition of 5 wt % HCPS into polymer/chloroform solution. It was shown that sorption on HCPS allowed for almost complete removal of tantalum compounds from PTMSP. The membrane made of PTMSP purified by HCPS demonstrated more stable transport characteristics compared to the membrane made of the initial polymer. HCPS has a complex effect on the aging process of PTMSP. The introduction of HCPS into the polymer matrix not only slowed down the physical aging of PTMSP, but also reduced chemical aging due to removal of active reagents.

## 1. Introduction

Membrane processes are among the most efficient technologies for the separation of gases and liquids due to low capital costs and small plant sizes compared to other separation methods that require solid or liquid adsorbents or processes such as distillation, which requires an energy-expensive phase transition [1,2,3,4]. Hydrophobic glassy polymers with a large fractional free volume (FFV) or polymers of intrinsic microporosity, such as disubstituted polyacetylenes, polybenzodioxane (PIM-1), and polynorbornenes, are considered promising membrane and sorbing materials for the separation of gases and liquids [5,6,7,8,9,10,11,12,13,14]. The increased interest in these polymers is based on their outstanding properties, in particular, the high fractional free volume, and as a result, high permeability. In membranes based on glassy polymers, a large proportion of the FFV is formed due to the rigidity of the polymer chains [2]. However, a significant disadvantage of glassy polymers is physical aging over time and, as a result, a significant decrease in the permeability coefficients. This poses a serious problem for the commercial application of these polymers [15,16,17,18,19,20]. The effect of physical aging on the gas permeability of various membrane materials is actively studied in the literature both from a theoretical and empirical point of view. For example, in the case of a poly[1-(trimethylsilyl)-1-propyne] (PTMSP) film with a thickness of 3 microns, nitrogen or helium permeability decreased more than twice during a 200 h test [20].

One of the simplest and most effective ways to reduce membrane aging is to introduce a filler phase to produce so-called mixed-matrix membranes (MMM) [21,22,23]. Molecular sieves [24], structures based on metal–organic frameworks (MOF) [25,26,27,28], porous aromatic frameworks (PAF) [16,29,30,31,32,33], hyper-crosslinked polystyrene (HCPS) [34,35,36,37,38], graphene, and graphene oxide (GO) [39] are used as such additives. MOF, such as zeolitic imidazolate frameworks (ZIF-7, -8), can increase the gas permeability, selectivity, and mechanical properties of membranes [26,27]. However, these improvements are short-lived, as these nanocomposites undergo accelerated physical aging [25]. PAF particles initially reduce the mechanical strength of glassy polymer films, but they stabilize over time and are mechanically stronger than aged mixed-matrix membranes impregnated with MOF [16]. The introduction of PAF into highly permeable glassy polymers improves their gas permeability characteristics due to an increase in the fractional free volume of the polymer and significantly suppresses physical aging due to a decrease in the mobility of macrochains as a result of partial sorption/incorporation of polymer segments into porous fillers. In particular, it was demonstrated that dense films based on PTMSP and PIM-1, with a thickness of 100 µm and containing 10 wt % PAF, showed only a 5–7% reduction in permeability to CO_2_ over 240 days, while unmodified PTMSP and PIM-1 showed a more pronounced reduction in gas transport by 38–62% [29]. Although functional additives such as MOF, PAF, and GO demonstrate good potential for creating MMMs with improved selectivity, permeability, and time stability, the production of these additives is expensive and difficult to scale up [40]. The best candidates for functional additives are HCPS obtained by simple crosslinking of aromatic monomers by the Friedel–Crafts reaction. They have a porosity similar to MOF and PAF. HCPS, first obtained by Davankov, Rogozhin, and Tsyurupa in the 1970s, represent a class of nanoporous materials with a wide range of practical and potential applications, such as sorption and gas separation, heterogeneous catalysis and chromatographic separation, and removal of metals from aqueous solutions [41,42,43,44,45,46]. For example, the introduction of HCPS in PIM-1 led not only to an increase in permeability, but also to a significant reduction in polymer aging and an increase in selectivity over time [36]. Earlier, PTMSP membranes filled with HCPS were produced for the first time by our group for gas separation and pervaporation tasks [38]. It was shown that the introduction of a small amount of the modifying component (0.5–1.0 wt %) into PTMSP matrix allows for the increase of the permeability coefficients of the material by 1.5 times for light gases (N_2_, O_2_, CO_2_, CH_4_). However, the question of the aging of these hybrid membranes over time was not studied thoroughly and is of great interest.

In this paper, a physical aging study was conducted on PTMSP membranes filled with commercial HCPS Macronet™ MN200 particles, with the control of the gas transport properties of the prepared membranes during up to 300 h annealing at 100 °C. In addition, for the first time HCPS was used as a sorbing agent for the deep purification of PTMSP from the polymerization catalyst and the production of PTMSP membranes with stable gas transport properties over time.

## 2. Materials and Methods

### 2.1. Materials and Reagents

PTMSP (SSP-070, lot 9D-35578) was purchased from Gelest, Inc. (USA). Macronet™ MN200 commercial sorbent (batch: 113X/18/15) was purchased from Purolite Ltd. Chloroform (chemical grade, Chimmed, Russia) was taken without further purification.

### 2.2. Preparation of PTMSP and PTMSP/HCPS Membranes

A solution of 1.5 wt % PTMSP in chloroform was used to produce the membranes. Dense PTMSP membranes were fabricated by casting PTMSP solution in chloroform on cellophane and subsequent drying for 200 h under ambient conditions at room temperature. The initial film diameter was 7.5 cm. The thickness varied in the range of 30–35 μm. The thickness of the membranes was measured using a Mitutoyo^®^ 293 Digimatic QuickMike electron micrometer with an accuracy of ±1 μm [4,31,38].

To produce MMM, we used an industrial sorbent of the biporous type (macro- and micropores) based on hyper-crosslinked polystyrene Macronet™ MN200 with specific surface area 1100 m^2^/g and density 0.48 g/cm^3^ as a filler [47]. The initial size of MN200 spherical pellets varied in the range of 300–1200 µm. The polymer dispersion was obtained by grinding the sorbent pellets in the swollen state in a vibration ball mill. The particle size of HCPS used as a membrane filler was close to 1 µm.

The PTMSP/HCPS casting suspensions with different filler content (0, 0.5, 1.0, 1.5, 2.0, 3.0, 5.0 and 10 wt %) were prepared by mixing two mixtures containing 1.5 wt % and 1.5 wt % of PTMSP and HCPS in chloroform, respectively. Before mixing with polymer solutions, the HCPS suspension was placed in the ultrasonic bath for 15 min. Then, prior to membrane casting, the PTMSP/HCPS suspensions were stirred using magnetic bar for 35 min and placed into the ultrasonic bath for another 15 min. The PTMSP/HCPS suspensions were cast onto a cellophane support and blanketed by a glass plate for slow evaporation of solvents at ambient conditions (about 200 h) until the constant weight of the samples was attained. The resulting thickness of the PTMSP/HCPS membranes varied in the range of 30–40 µm.

### 2.3. Particle Size Distribution

The size distributions of HCPS particles obtained after grinding the initial commercial MN200 samples were determined by dynamic light scattering on a Malvern Zetasizer Nano analyzer (Malvern Panalytical Ltd., Malvern, UK) [33]. The test samples were prepared by suspending the particles in chloroform (0.06 g of a sample in 10 mL of solvent).

### 2.4. PTMSP Molecular Weight

Gel-permeation chromatography (GPC) analysis of the polymers was performed on a Waters chromatograph system with a differential refractometer (Chromatopack Microgel-5, chloroform as the eluent, flow rate 1 mL/min) [9,10]. Molecular mass and polydispersity were calculated by standard procedure relative to monodispersed polystyrene standards.

### 2.5. Membrane Characterization

Scanning electron microscopy (SEM) was used to characterize the structure and morphology of the membranes. SEM was carried out on a Thermo Fisher Phenom XL G2 Desktop SEM (USA). Cross-sections of the membranes were obtained in liquid nitrogen after preliminary impregnation of the specimens in isopropanol. A thin (5–10 nm) gold layer was deposited on the prepared samples in a vacuum chamber (≈0.01 mbar) using a desktop magnetron sputter “Cressington 108 auto Sputter Coater” (UK). The accelerating voltage during images acquisition was 15 kV. Further image analysis and determination of the selective layer thickness was carried out using the Gwyddion software (ver. 2.53).

The elemental composition of the initial and purified PTMSP membranes was determined by wavelength dispersive X-ray fluorescence analysis (WDXRF), carried out on an ARL PERFORM’X (Thermo Fischer Scientific) sequential X-ray fluorescence spectrometer using a rhodium tube [48]. Up to 79 elements were analyzed and the percentage composition of the sample was calculated by the UniQuant program to a relative error of 5%.

### 2.6. Gas Transport Parameters

Single gas permeation measurements (N_2_, O_2_, CO_2_, and CH_4_) were carried out at a temperature of 30.0 ± 0.1 °C and at a feed pressure of 0.1–0.8 bar, using a constant volume/variable pressure experimental setup (GKSS Time-Lag Machine) [9,49]. The measurements were performed for the as-cast PTMSP and PTMSP/HCPS membranes and for the same membranes after their annealing in air at 100 °C for 50, 100, and 300 h. Permeability coefficient *P* expressed in Barrer (1 Barrer = 10^−10^ cm^3^(STP)⋅cm⋅cm^−2^⋅s^−1^⋅cmHg^−1^) was estimated by the linear extrapolation of experimental data to zero trans-membrane pressure. Diffusion coefficient *D* was estimated using the time-lag method as *D* = *l^2^/6θ*, where *l* is the membrane thickness, and *θ* is the experimental time lag before the attainment of the steady state permeation regime. Solubility coefficient *S* was indirectly evaluated in terms of the solution–diffusion permeation model: *S* = *P/D*. Ideal selectivity of the pair of gases was calculated as the ratio of permabilities of individual gases.

### 2.7. Sorption of the Polymerization Catalyst

PTMSP was purified by adding a sample of the initial commercial HCPS MN200 (5 wt %) in a solution of PTMSP in chloroform and left to mix for 24 h. The resulting suspension was then filtered several times under pressure through commercial microfiltration hydrophobic membrane MFFK-1 consisting of fluoroplastic F42L (copolymer of tetrafluoroethylene and vinylidene fluoride) on polypropylene support (Vladipor, Russia) to completely remove HCPS particles from the suspension.

## 3. Results and Discussion

### 3.1. PTMSP Membranes Filled with HCPS

**Figure 1:**
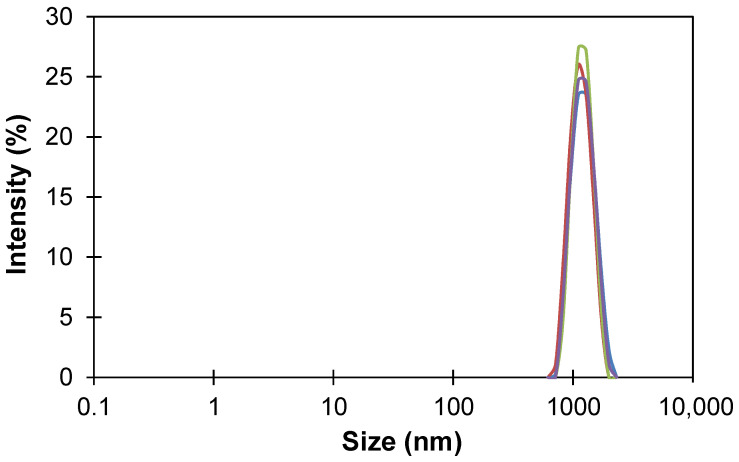
Size distribution of HCPS MN200 in chloroform.

Hyper-crosslinked polymers were first produced by Davankov, Rogozhin, and Tsyurupa and are now produced in volume by Purolite Corporation. The commercial hyper-crosslinked polystyrene sorbent Macronet^TM^ MN200 was chosen as a modifying additive since it is the most used sorbent of the Macronet^TM^ family for the separation of volatile organic compounds from aqueous solutions and it also has a high surface area (1100 m^2^/g) [47]. The initial size of MN200 spherical pellets varied in the range of 300–1200 µm; therefore, the sorbent pellets were previously milled in a swollen state in a vibration ball mill to produce MMM. Figure 1 shows functions of the normal size distribution of milled MN200 particles in the dispersion in chloroform. The average particle size was determined by the maximum of intensity, and thus the HCPS particle size after milling was 1.2 µm.

A commercial PTMSP polymer purchased from Gelest, Inc. was used to produce the membranes. According to the results of gel-permeation chromatography analysis, we determined the molecular mass of PTMSP, which was about 250 × 10^3^ g/mol; a similar molecular weight for this commercial polymer was also mentioned in the work of Claes [50].

**Figure 2:**
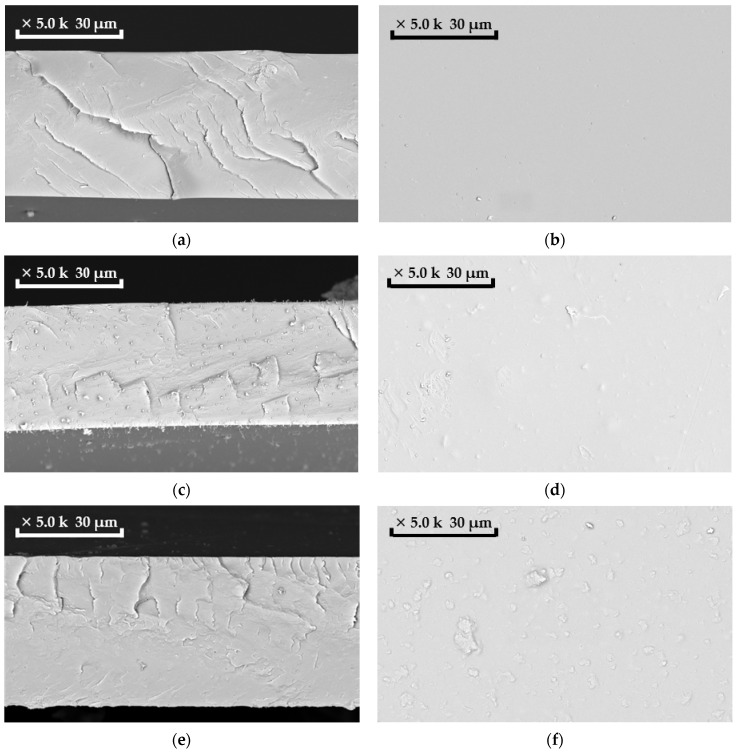

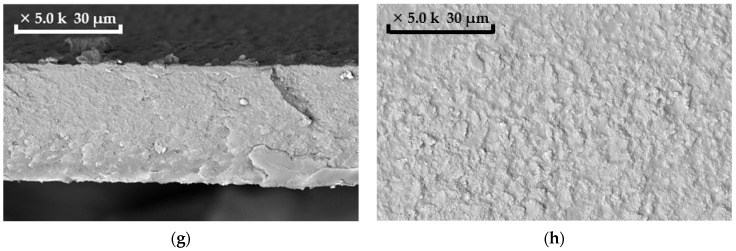
SEM images of membranes: (**a**) cross-section of PTMSP membrane, magnification 5000×; (**b**) top view of PTMSP membrane, magnification 5000×; (**c**) cross-section of membrane with 0.5 wt % HCPS content in PTMSP, magnification 5000×; (**d**) top view of the membrane with 0.5 wt % HCPS content in PTMSP, magnification 5000×; (**e**) cross-section of the membrane with 2.0 wt % HCPS content in PTMSP, magnification 5000×; (**f**) top view of the membrane with 2.0 wt % HCPS content in PTMSP, magnification 5000×; (**g**) cross-section of the membrane with 10 wt % HCPS content in PTMSP, magnification 5000×; (**h**) top view of the membrane with 10 wt % HCPS content in PTMSP, magnification 5000×.

By mixing PTMSP solution and HCPS suspension in chloroform, we produced hybrid membranes with a variable content of HCPS particles (from 0.5 to 10 wt %). The morphology of MMM reflects the structural features of the polymer matrix system with impregnated particles and is one of the main factors determining the physical properties of such hybrid membranes. The distribution of HCPS particles on the surface and in the volume of the membranes was studied by scanning electron microscopy (SEM) (Figure 2). SEM studies of hybrid membranes have shown that such membranes have a uniform distribution of HCPS particles over the matrix volume, but HCPS particles located on the membrane surface concentrate and form individual agglomerates up to 15 µm. In addition, with an increase in HCPS additive, a loose structure of PTMSP polymer matrix is formed; this can be clearly seen on the cross-section of the PTMSP membrane with an addition of 10 wt % HCPS (Figure 2g).

**Figure 3:**
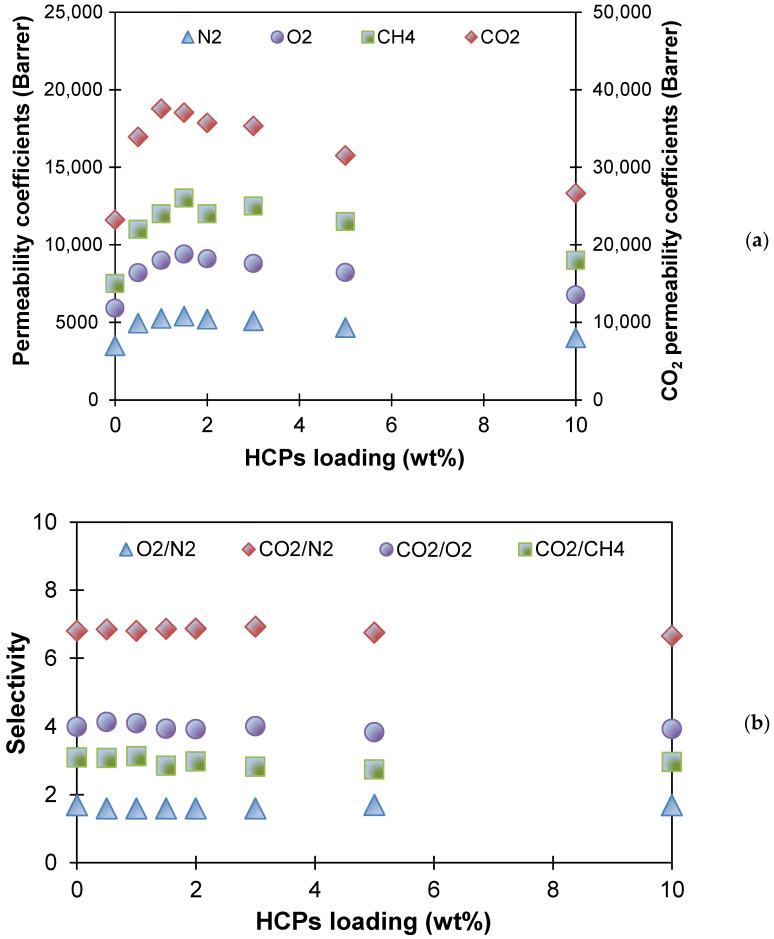
Dependencies of (**a**) permeability coefficients for light gases (N_2_, O_2_, CO_2_, CH_4_) and (**b**) O_2_/N_2_, CO_2_/N_2_, CO_2_/O_2_, and CO_2_/CH_4_ ideal selectivities on HCPS loading.

The next step was to study the gas transport properties of the produced membranes. The permeability and diffusion coefficients of light gases (N_2_, O_2_, CO_2_, CH_4_) in MMM materials of various compositions were determined by the Deynes–Barrer method. Figure 3 shows the dependences of the permeability coefficients of various penetrants (Figure 3a) and the gas pair selectivity of O_2_/N_2_, CO_2_/N_2_, CO_2_/O_2_, and CO_2_/CH_4_ (Figure 3b) in the HCPS concentration range of 0–10 wt %. It is shown that the permeability coefficients for all penetrants increased by 50–60%, with an increase of MN200 concentration in the membrane material from 0 to 1 wt %; thus, the permeability coefficient for nitrogen increased from 3000 to 5300 Barrer, and for carbon dioxide from 20,800 to 37,600 Barrer. Gas permeability gradually decreased with an increase in the HCPS concentration above 1 wt %. It is worth noting that the ideal separation selectivity was practically constant (Figure 3b). The obtained values of selectivity for O_2_/N_2_, CO_2_/N_2_, CO_2_/CH_4_, and CO_2_/O_2_ gas pairs coincided with the previously published values in the literature for PTMSP membranes; for example, the selectivity for O_2_/N_2_ pair was 1.7 [51,52]. The increase in the gas permeability coefficients of PTMSP when it is modified with HCPS was associated with the effect of increasing the FFV of the material, since hyper-crosslinked polystyrene has a large FFV (more than 30%). This fact correlates with the literature data, wherein the inclusion of various MOF, PAF, and HCPS additives in glassy polymers (PIM-1 and PTMSP) also leads to an increase in permeability with a selectivity similar to or close to the control sample [29,30,31,38,53].

**Figure 4:**
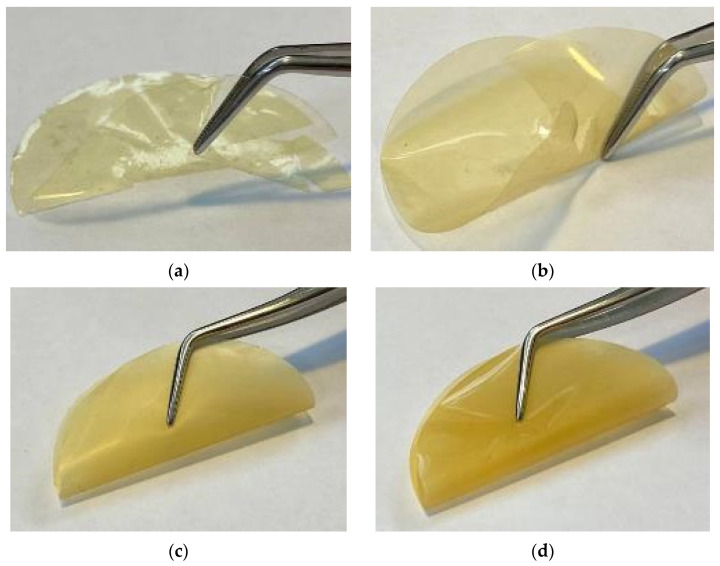
PTMSP (**a**), PTMSP/HCPS 1.0 wt % (**b**), PTMSP/HCPS 2.0 wt % (**c**) and PTMSP/HCPS 5 wt % (**d**) films after 400 h of annealing at 100 °C.

It is known that the polymer–additive interaction can lead not only to increased values of permeability or selectivity, but also to improved mechanical properties and stability of the membranes over time due to reduced physical aging. Therefore, the resulting PTMSP membranes with different HCPS loading were annealed at 100 °C for up to 400 h to facilitate structural rearrangements of the macrochains and thus to accelerate physical aging. The gas permeability of the samples was measured after 50, 100, and 300 h of heat treatment. Previously, the effective use of temperature–time superposition was shown by studying the physical aging of unmodified PTMSP films, as well as films with embedded filler particles [31,54,55,56]. It was shown that unmodified dense PTMSP membranes do not withstand prolonged high temperature exposure in air and break down into large fragments after 510 h of thermal annealing, while PTMSP membranes filled with PAF-11 demonstrate excellent mechanical properties [31]. In our annealing experiments, unmodified PTMSP sample (not filled with HCPS particles) collapsed after 400 h of annealing (Figure 4a). Moreover, the membrane became so fragile that it collapsed into small pieces at even the slightest touch. In addition to the initial PTMSP membranes, the PTMSP membranes containing 0.5 and 1.0 wt % of HCPS also failed to withstand 400 h of annealing and broke into large fragments (Figure 4b). PTMSP membranes containing 2.0 and 5.0 wt % of HCPS remained intact, demonstrating good flexibility and mechanical strength in a simple membrane bending test (Figure 4c,d). These results confirm that the addition of porous fillers improves the mechanical properties of polymer membranes.

**Figure 5:**
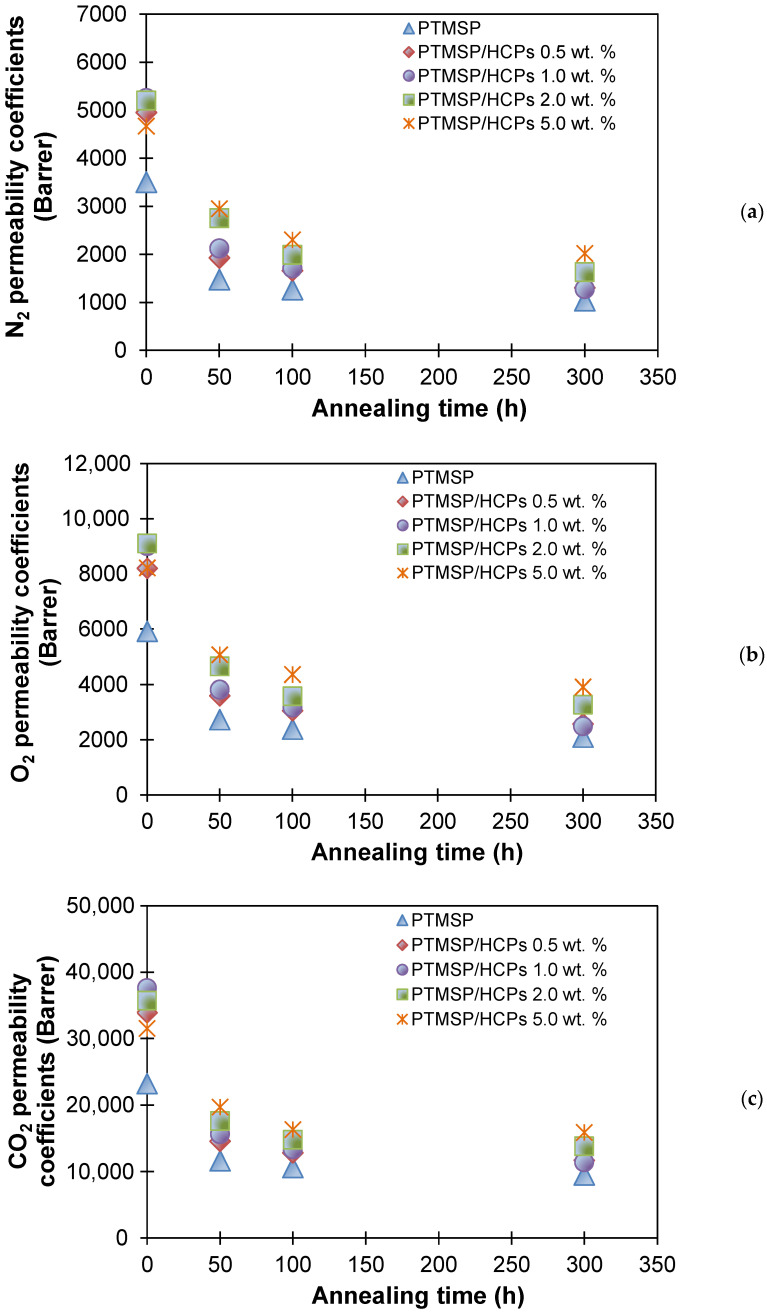
N_2_ (**a**), O_2_ (**b**), and CO_2_ (**c**) permeability coefficients for PTMSP and PTMSP/HCPS membranes containing 0.5, 1.0, 2.0, and 5.0 wt % of HCPS plotted against annealing time at 100 °C.

Figure 5 shows the dependences of N_2_, O_2_, and CO_2_ permeability coefficients on the annealing time for the membranes cast from different suspensions. As can be seen, all membranes showed a decrease in permeability coefficients during the 300 h annealing process; however, samples filled with HCPS had higher transport characteristics than the initial PTMSP throughout the annealing. With an increase in HCPS addition, the gas permeability of the membranes quickly reached a stable value during the annealing. This was especially noticeable for the PTMSP membrane with 5.0 wt % HCPS addition, which showed the maximum permeability coefficients after 300 h of annealing for all of the studied gases.

Changes in the coefficients of relative gas permeability (P_300_/P_0_) and ideal selectivity during annealing are summarized in Table 1. Previously, it was shown that a small inclusion of HCPS (up to 1.0 wt %) in PTMSP allows one to prepare membranes with improved gas permeability (Figure 3) in comparison with the initial PTMSP membrane, but with similar values of ideal gas selectivity. However, during annealing, the gas permeability coefficients of the PTMSP/HCPS films (0.5 and 1.0 wt %) decreased by 65–75%. After annealing for 300 h, the gas permeability coefficients of the unmodified PTMSP membrane decreased by 60–71%. At the same time, the lowest reduction in gas permeability coefficients (50–57%) during 300 h of annealing was found for PTMSP/HCPS 5.0 wt % sample, and this sample also showed the fastest time to stable permeability coefficients. This effect can be attributed to the fact that rigid HCPS chains better constrain the mobility of PTMSP macrochains with the addition of more than 1 wt % of HCPS in PTMSP; the permeability of the hybrid membrane is averaged over the polymer and dispersed phases. It is also worth noting that during the annealing of the membranes, the ideal selectivity for gas pairs O_2_/N_2_, CO_2_/N_2_, CO_2_/O_2_ increased in the case of all membranes.

**Figure 6:**
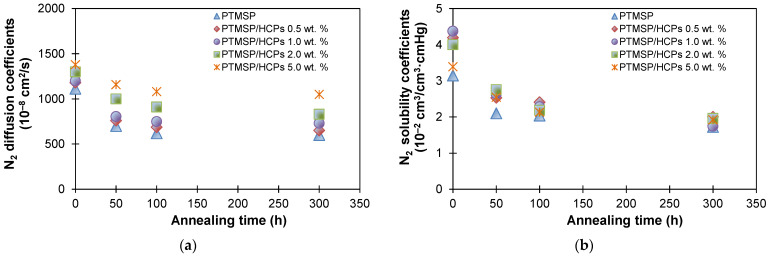

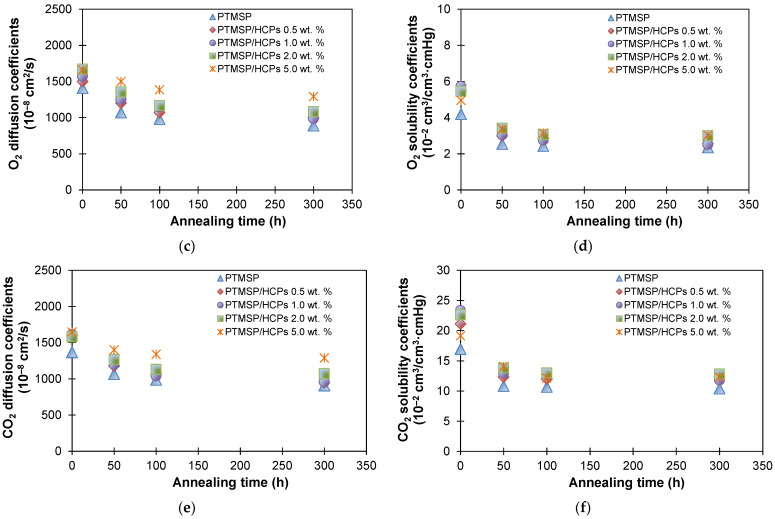
Diffusion coefficients and solubility coefficients of N_2_ (**a**,**b**), O_2_ (**c**,**d**), and CO_2_ (**e**,**f**) in PTMSP/HCPS membranes containing 0, 0.5, 1.0, 2.0, and 5.0 wt % of HCPS plotted against the time of annealing at 100 °C.

Figure 6 shows the diffusion coefficient and solubility coefficient for N_2_, O_2_, and CO_2_ in PTMSP with different HCPS content, depending on the annealing time. As can be seen, the introduction of porous fillers led to an increase in the diffusion coefficient and solubility. The porous structure of HCPS is characterized by a sufficiently large specific surface area and regular pores, which are attractive for the intrusion of PTMSP chain fragments. Thus, the maximum gas permeability coefficient of PTMSP/HCPS at 5.0 wt % after 300 h of annealing can be explained by the higher loading of porous fillers, which are not subject to aging, unlike PTMSP, and which facilitate the transport of gas through the membrane. Characteristics of PTMSP membranes with lower (less than 2 wt %) HCPS content approached the characteristics of the initial PTMSP during annealing (Figure 6). Meanwhile, all the membranes showed similar behavior during annealing, which is a decrease in the diffusion and solubility coefficients.

### 3.2. PTMSP Purification from Polymerization Catalyst

HCPS has excellent sorption properties and is used as a sorbent in the separation of organic and inorganic (for example, metals) components from aqueous solutions [44,45,57]. PTMSP membranes filled with HCPS for gas separation and pervaporation tasks were produced for the first time by our group earlier [38]. It was found that the initial (unmodified) membrane contains tantalum inclusions, which are evenly distributed throughout the entire volume of the membrane. These inclusions remain as a result of insufficient purification of PTMSP from the polymerization catalytic system (TaCl_5_ with triisobutylaluminium) used in the process of polymer synthesis [51,58]. At the same time, the standard technology for cleaning PTMSP from excess monomer, oligomers, and spent catalysts is the repeated reprecipitation of the polymer in excess (4–6 times) of methanol, followed by its drying in vacuum. In the case of PTMSP membranes filled with HCPS, tantalum compounds were found only in the HCPS particles. It was hypothesized that the introduction of HCPS can purify the PTMSP matrix from the residues of the catalytic system, which may also affect the stability of the characteristics of hybrid membranes over time. However, the effect of the presence of catalyst compounds on the transport and physical properties of the PTMSP membrane has not yet been studied.

In order to check this hypothesis, firstly, we determined the elemental composition of a commercial PTMSP membrane using wavelength dispersive X-ray fluorescence analysis (WDXRF) (Table 2). It was found that the matrix of the membrane, beside silicon (25.2 wt %) and hydrocarbon groups (73.3 wt %), contained 0.86 wt % of tantalum. However, an interesting discovery was that, along with tantalum, palladium was also found in a slightly lower concentration (0.53 wt %). It should be noted that the presence of Pd, both in the PTMSP solution and in the membrane, is indirectly indicated by their yellow-brown color, which is characteristic of Pd salts. The presence of a significant amount of Pd in PTMSP raises a serious question; on the basis of the literature data, tantalum and niobium chlorides or bromides are usually used as catalysts in the synthesis of PTMSP, as well as triisobutylaluminium as a co-catalyst [8,51,58,59,60]. Most likely, Pd was used in the preparation of the monomer 1-(trimethylsilyl)-1-propyne (TMSP) and subsequently transferred into PTMSP.

Secondly, we decided to purify PTMSP from the residues of the catalytic compounds by adding 5 wt % of unmilled commercial MN200 HCPS pellets in a polymer solution in chloroform. The polymer suspension with HCPS was stirred for 24 h, and then the HCPS pellets were filtered out under pressure using the MFFK-1 microfiltration membrane. The filtrate had a polymer concentration slightly lower than the initial one (1.2 vs. 1.5 wt %). Most likely, the HCPS absorbed some of the polymer, and the other part of the polymer remained on the walls of the filter cell and the microfiltration membrane. Compared to the standard technology used for the purification of aqueous solution from organic and inorganic compounds, when a long column filled with HCPS was used, filtration through a membrane resulted in lesser polymer losses and higher efficiency. The color of the PTMSP solution in chloroform after filtering from HCPS did not change. A film was cast from the purified solution and subsequently examined by the WDXRF method (Table 2). It can be seen that with the use of HCPS, it was possible to almost completely clear the membrane from tantalum, but the palladium content decreased only slightly. This fact once again indicates that Pd was most likely included in the PTMSP at the monomer production stage and was firmly bound to the polymer structure.

**Figure 7:**
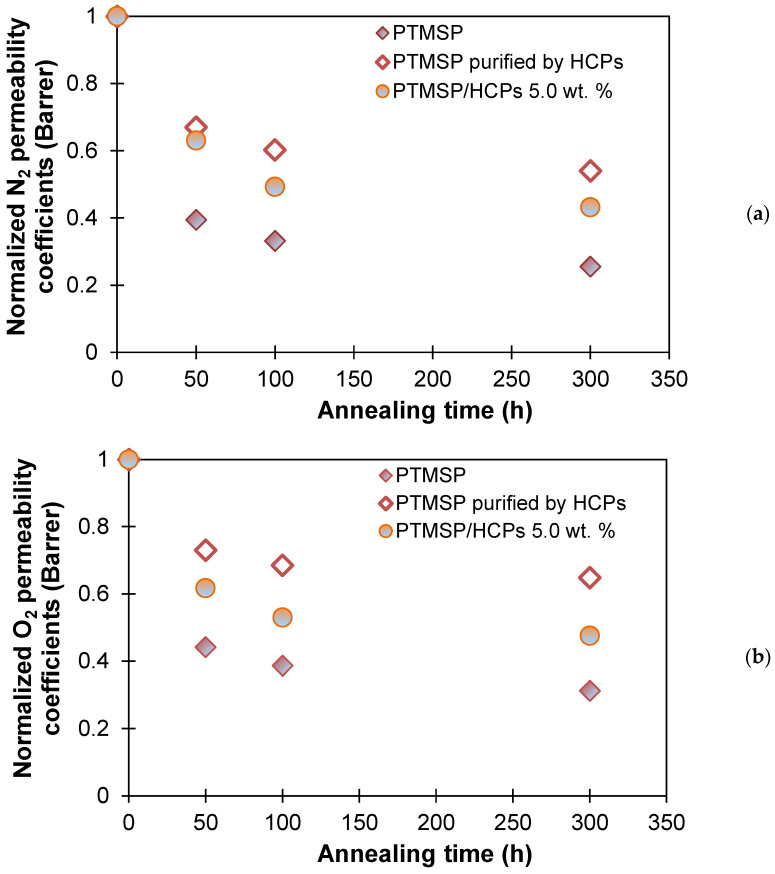

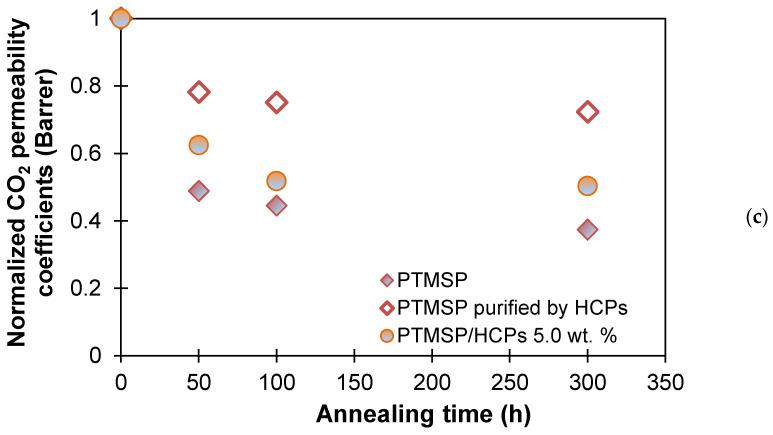
N_2_ (**a**), O_2_ (**b**), and CO_2_ (**c**) permeability coefficients for PTMSP membrane (painted diamond), PTMSP purified by HCPS (unpainted diamond), and PTMSP/HCPS 5.0 wt % (painted circle) against annealing time at 100 °C.

For the study of physical aging, we obtained two PTMSP membranes with as close as possible thickness from the initial polymer solution and purified them from the residues of polymerization catalysts by HCPS. N_2_, O_2_, and CO_2_ permeability was measured in fresh samples, and then, as in the case of hybrid PTMSP/HCPS membranes, the samples were subjected to thermal annealing at 100 °C for 300 h (Figure 7). The gas permeability of the samples was measured after 50, 100, and 300 h of heat treatment. Figure 7 shows the normalized permeability coefficient with respect to the initial values for the unmodified PTMSP membrane; PTMSP purified by HCPS; and, as a comparison, PTMSP/HCPS 5.0 wt %, as it demonstrated the most stable gas transport properties among MMM during the annealing process. The initial values of the permeability coefficients for the unmodified PTMSP membrane (P_CO2_ = 26,100 Barrer) were 1.2 times higher compared to the PTMSP purified by HCPS (P_CO2_ = 21,100 Barrer). However, after just 50 h of annealing, the gas transport properties were reduced by 52–60% for the unmodified and not purified from polymerization catalyst membrane (Figure 7). At the same time, the membrane made from PTMSP purified by HCPS showed a decrease of only 22–32% after 50 h of annealing. As can be seen from Figure 7, the PTMSP membrane purified by HCPS demonstrated the most stable gas transport properties during the thermal annealing process, even in comparison with the hybrid PTMSP/HCPS 5 wt % membrane. The stabilization of the gas transport properties during annealing of the membrane made from PTMSP purified from the residues of the catalysts was most likely due to the initially denser packing of the macrochains due to the absence of tantalum residues, which affects the reduction of physical aging during annealing. However, as with PTMSP, PTMSP/HCPS 0.5 wt %, and PTMSP/HCPS 1.0 wt % membranes described above, these samples (non-purified and purified by HCPS membranes) also collapsed after 400 h of annealing. Thus, the purification of the polymer from the residues of the catalytic system provides more stable gas transport properties of the membrane over time, but it does not improve the mechanical stability of the material.

**Figure 8:**
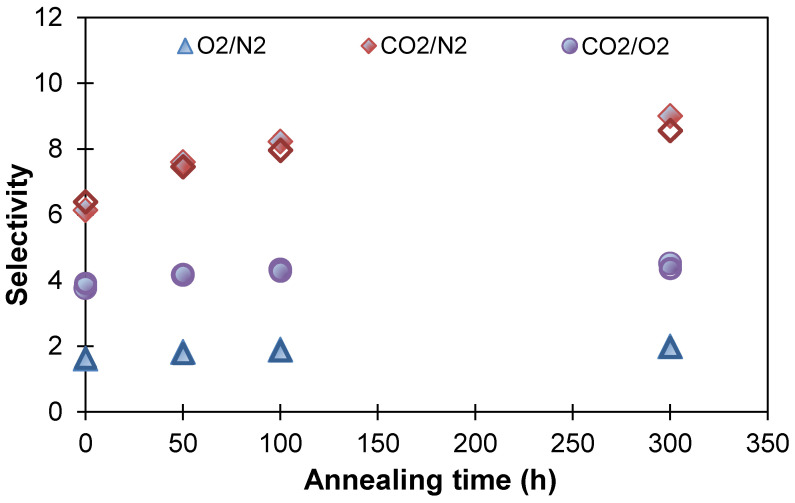
Changes in ideal selectivity for the initial PTMSP membrane (painted markers) and membrane from PTMSP purified by HCPS (unpainted markers) after annealing at 100 °C for 300 h.

As for the ideal selectivity for O_2_/N_2_, CO_2_/N_2_, and CO_2_/O_2_ gas pairs, it increased during annealing for both investigated PTMSP membranes (unmodified and purified) and reached stationary values during annealing (Figure 8). The membrane from PTMSP purified by HCPS showed slightly lower values than the initial PTMSP membrane, in terms of the selectivity for the CO_2_/N_2_ gas pair. The purification of the polymer from the residues of the catalysts makes it possible to produce membranes with improved stability of gas transport properties over time. It can be concluded that in the case of HCPS, as in the case of other additives with high porosity and good sorption properties (MOF, PAF), the effect of reducing aging occurs not only due to the introduction of PTMSP chains into the pores of the additive, which provides resistance to polymer relaxation, but also due to the purification of PTMSP from the residues of the catalysts.

## 4. Conclusions

The temperature–time superposition principle was used to study the physical aging of the initial PTSMP, PTSMP with porous hyper-crosslinked polystyrene (HCPS), and PTMSP purified from residuals of polymerization catalyst. The industrial sorbent Purolite Macronet™ MN200, with a high sorption capacity for organic solvents, was chosen as HCPS. The gas transport characteristics of dense PTMSP membrane with a thickness of 30–40 µm containing 0, 0.5, 1.0, 2.0, and 5.0 wt % HCPS were studied, as-cast and after annealing at 100 °C for 50, 100, and 300 h. It was shown that the introduction of a small amount of HCPS (up to 1.0 wt %) in the PTMSP matrix allowed 50–60% increase of the permeability coefficients of the material for light gases (N_2_, O_2_, CO_2_, CH_4_) with stable ideal selectivities for gas pairs O_2_/N_2_, CO_2_/N_2_, CO_2_/O_2_, and CO_2_/CH_4_. After annealing for 300 h, the gas permeability coefficients of the unmodified PTMSP membrane decreased by 60–71%. At the same time, the lowest reduction in gas permeability coefficients (50–57%) during annealing was found for PTMSP with 5.0 wt % HCPS, which eventually showed the maximum permeability coefficients for all penetrants. It was demonstrated that the introduction of more than 1 wt % of HCPS fillers into PTMSP significantly improved the mechanical stability of materials during annealing, whereas unmodified PTMSP and PTMSP containing 0.5 and 1.0 wt % HCPS could not withstand heat treatment in air at 100 °C for 400 h and broke up into fragments. Gas transport characteristics of a membrane made from PTMSP containing 5 wt % HCPS became stable after a short annealing time (200–300 h). This effect can be attributed to the fact that rigid HCPS chains better constrain the mobility of PTMSP macrochains with the addition of more than 1 wt % of HCPS; the permeability of the hybrid membrane is averaged over the polymer and dispersed phases.

It was found that HCPS adsorbs a residual polymerization catalyst on the basis of tantalum compounds from PTMSP. On the basis of these findings, we developed a new method for purification of PTMSP from the polymerization catalyst, which included the addition of 5 wt % HCPS pellets into a polymer solution in chloroform and its subsequent filtration. It was shown that sorption on HCPS allowed for almost complete removal of tantalum compounds from PTMSP. Investigation of N_2_, O_2_, and CO_2_ permeability of the initial and purified membranes during the annealing (100 °C for up to 300 h) demonstrated that a membrane from PTMSP purified by HCPS had high permeability coefficients and the most stable transport characteristics. Therefore, HCPS was shown to have a complex effect on the aging process of PTMSP. The introduction of HCPS into the polymer matrix not only slowed down the physical aging of PTMSP, but also reduced chemical aging due to removal of active reagents.

## Figures and Tables

**Table 1:** Changes in relative gas permeability coefficients and ideal selectivity after annealing at 100 °C for 300 h.

Membranes	Decrease in Gas Permeability Coefficient, %			Selectivity					
				O_2_/N_2_		CO_2_/N_2_		CO_2_/O_2_	
	N_2_	O_2_	CO_2_	Before	After	Before	After	Before	After
PTMSP	71	65	60	1.7	2.0	6.7	9.2	4.0	4.5
PTMSP/HCPS 0.5 wt %	73	68	65	1.7	2.0	6.8	9.1	4.1	4.5
PTMSP/HCPS 1.0 wt %	75	72	69	1.6	2.0	6.9	8.9	4.1	4.5
PTMSP/HCPS 2.0 wt %	69	64	61	1.6	2.0	6.8	8.7	4.0	4.3
PTMSP/HCPS 5.0 wt %	57	52	50	1.6	1.9	6.8	8.2	3.9	4.2

**Table 2:** The elemental composition of the initial PTMSP membrane, and the membrane produced after purification of PTMSP solution by HCPS.

Element	Content in the Initial PTMSP Membrane, 31 µm, wt%	Content in Membrane after Purification of PTMSP Solution by HCPS, 34 µm, wt%
Si	25.2	25.3
Ta	0.86	0.06
Cl	0.07	0.02
Al	0.006	0.001
Pd	0.53	0.42

## Data Availability

The data presented in this study are available on request from the corresponding author.
